# O Twitter (X) como Ferramenta de Comunicação e Educação para Cardiologistas Brasileiros: Perfil, Influência e Desafios

**DOI:** 10.36660/abc.20230694

**Published:** 2024-11-27

**Authors:** Aline Goneli de Lacerda, Luana de Oliveira Ribas, Estephany de Jesus, Ronaldo Ferreira de Araújo, Thaiane Moreira de Oliveira, Claudio Tinoco Mesquita

**Affiliations:** 1 Universidade Federal Fluminense Niterói RJ Brasil Universidade Federal Fluminense, Niterói, RJ – Brasil; 2 Universidade Federal de Alagoas Maceió AL Brasil Universidade Federal de Alagoas, Maceió, AL – Brasil

**Keywords:** Redes Sociais Online, Cardiologistas, Comunicação em Saúde

## Abstract

**Fundamento:**

O Twitter (recentemente renomeado X) é uma mídia social popular que pode ser usada para a comunicação em saúde, mas há poucos estudos sobre o perfil dos cardiologistas brasileiros na plataforma. Objetivos: Identificar o perfil dos cardiologistas brasileiros no Twitter (X), sua rede de influência e alcance, e a forma de apresentação da sua biografia.

**Métodos:**

Foram analisadas 1083 contas de cardiologistas brasileiros criadas entre 2006 e 2021. Os dados foram coletados usando a ferramenta FollowerWonk e analisados com o software IRAMUTEQ.

**Resultados:**

A maioria dos perfis foi de homens (76,5%) e 0,8% era institucional. A maioria dos perfis tem até 100 seguidores (71%) e baixa autoridade social (81,8%). Os 20 perfis de maior influência são de homens (80%), com alta concentração na região sudeste (68%).

**Conclusões:**

Os cardiologistas brasileiros preferem uma comunicação pessoal e direta nas redes sociais, em vez de uma representação institucional. Há uma disparidade de gênero entre os cardiologistas brasileiros no Twitter (X), com predominância de homens. Os cardiologistas brasileiros com maior influência online são homens, com alta concentração na região sudeste. Novos estudos devem ser realizados sobre o tema para verificar o impacto destas características na população.

## Graphical abstract

**Figura Central:**
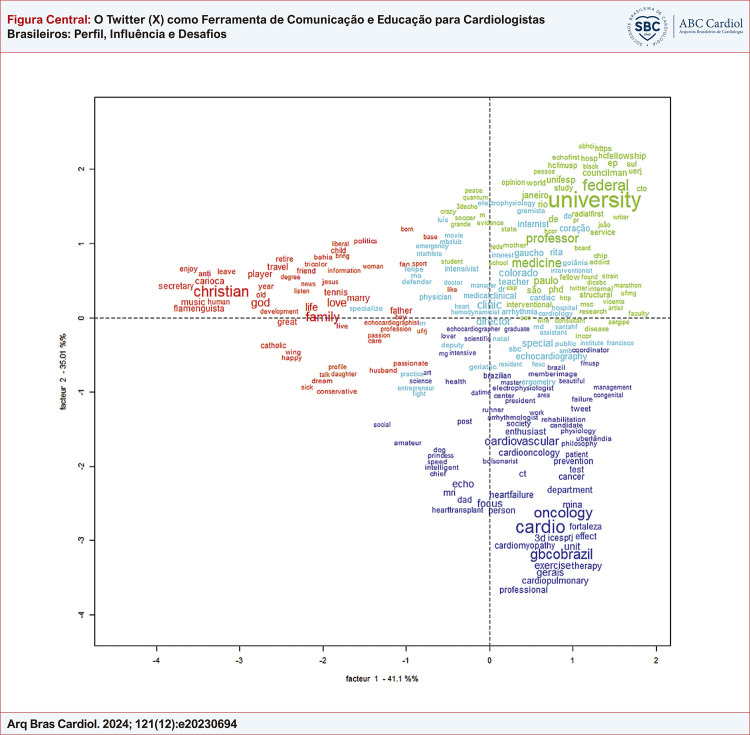
O Twitter (X) como Ferramenta de Comunicação e Educação para Cardiologistas Brasileiros: Perfil, Influência e Desafios

Análise fatorial de correspondência demonstrando as palavras mais utilizadas e suas associações gerada no Iramuteq a partir dos Tweets dos cardiologistas brasileiros.

## Introdução

Desde que a Internet se tornou um local comum para divulgar e acessar informações de saúde,^1^as mídias sociais têm sido um espaço cada vez mais importante em que profissionais de saúde e acadêmicos compartilham resultados de pesquisas e informações científicas e fortalecem os laços com os pacientes.^2^

O Twitter (X) é atualmente a mídia social mais usada para comunicação em saúde.^3^ Compartilhar informações no Twitter (X) pode criar uma atmosfera comunicativa e colaborativa para pacientes, médicos e pesquisadores e até mesmo melhorar a qualidade do atendimento. Devido aos recursos da plataforma que permitem uma comunicação conversacional interpessoal,^4-6^ os tweets têm o potencial de capitalizar as mídias sociais para ampliar o alcance das mensagens de saúde.^7^

O Twitter (X) ganhou um papel importante como fórum acadêmico, especialmente por sua natureza de *microblog*, que permite interações diretas entre diversos especialistas em tempo real e rápido.^8^ Cerca de 20% dos artigos no PubMed são tweetados pelo menos uma vez e isso pode aumentar as chances de citação.^5^ No entanto, apesar dessa conquista científica, muito poucos médicos e cientistas se envolvem com o Twitter (X) rotineiramente,^9^ conforme indicado em uma pesquisa que apenas 238 de 1,500 cardiologistas (16%) possuíam contas no Twitter (X). Embora existam inúmeras explicações potenciais para essa baixa adoção entre a comunidade científica, preocupações importantes em relação à promoção de pontos de vista infundados, manipulação de dados, uso ineficiente do tempo e privacidade do paciente provavelmente sejam os principais contribuintes.

Conforme observado por Ferguson et al.,^10^ houve um aumento no percentual de profissionais da área cardiovascular, incluindo periódicos e associações, que utilizam o Twitter (X) para interagir com outras pessoas e trocar ideias. A avaliação do âmbito e do impacto da pesquisa em saúde e da prática médica nas redes sociais pode fornecer informações sobre melhores estratégias para promover a utilização das redes sociais. Embora alguns pesquisadores discutam o perfil profissional de pesquisadores e profissionais de saúde nas mídias sociais de diferentes países,^3,10,11^ ainda não há estudos focados no contexto brasileiro.

Portanto, o objetivo desta pesquisa é identificar quem são os cardiologistas brasileiros presentes no Twitter (X), sua rede de influência e alcance, e como eles se apresentam em sua bio descrição. Entendemos que a utilização das mídias digitais pelos profissionais da cardiologia é uma forma de construção de autoridade e capital social importantes para entender como a área pode ser apresentada no microblog.

## Métodos

A presente pesquisa se caracteriza como exploratória de abordagem quantitativa, descritiva com intuito de identificar a presença, visibilidade e influência *online* de cardiologistas brasileiros no Twitter (X).

### Coleta de dados

As biografias dos usuários do Twitter (X) foram examinadas com a ferramenta baseada na web FollowerWonk (https://moz.com/followerwonk) usando as palavras-chave ‘cardiologist’ OR ‘cardiologista’ em dezembro de 2022. O Followerwonk tem a capacidade de visualizar redes do Twitter (X) geograficamente, comparar diferentes contas de usuários e analisar melhor o conteúdo dos Tweets de regiões específicas.^12^

Todos os dados do perfil, incluindo o Social Authority Score (SAS), foram exportados para uma planilha de banco de dados onde foi realizada a análise estatística descritiva. The SAS é uma escala de influência do Twitter (X) (1–100) que considera indicadores-chave de desempenho, como número de seguidores, menções de usuários, número de retuítes (*retweets*, ou RT) e engajamento das publicações dos usuários no Twitter (X).^13^

Os critérios de exclusão dos perfis foram: (a) perfis pessoais ou institucionais não pertencentes a cardiologistas; (b) Contas que não estivessem em português ou em inglês; (c) Usuário inativo (nenhum tweet postado nos últimos 6 meses); (d) Localização de usuário fora do Brasil ou usuário sem vínculo com instituição brasileira; (e) Perfis restritos; e (f) Perfis sem fotos.

### Análise dos dados

As variáveis de análise consideradas na pesquisa foram: (i) número de perfis identificados como cardiologistas brasileiros e data de criação da conta; (ii) URLs disponíveis na descrição dos perfis; (iii) número de seguidores de cardiologistas brasileiros (média, desvio padrão); (iv) top 100 perfis de autoridade social; (v) correlação das 100 principais localizações geográficas e Autoridade Social; (vi) desigualdades de gênero e raça relacionadas ao uso da Cardiologia no Twitter (X), e; (vii) tópicos mais comuns tweetados.

Os dados de descrição de bio de cada usuário foram extraídos e organizados em uma planilha csv. Para o processamento dos dados, utilizou-se o software IRAMUTEQ (*Interface de R pour lês Analyses Multidimensionnelles de Textes et de Questionnaires*). Trata-se de um programa livre de linguagem em R, e que permite processamento e análises estatísticas de textos produzidos.^14^ Para análise dos conteúdos textuais das bios foram utilizadas as técnicas de Classificação Hierárquica Descendente (CHD) e Análise Fatorial de Correspondência (AFC), que permitem sua identificação por meio de um arquivo textual único, devidamente configurado.

## Resultados

As características descritivas extraídas dos perfis de cardiologistas brasileiros identificados no Twitter (X) indicam que as 1083 contas analisadas foram criadas entre os anos de 2006 e 2021. O Gráfico 1 apresenta a distribuição das contas pelo ano de criação.

Houve baixa adesão nos primeiros anos do *microblog*, sendo observado um pico de contas criadas em 2009 (n= 191) e 2010 (n=125) havendo uma progressiva queda nos anos seguintes. A partir de 2017, observamos nova retomada, com destaque aos anos de 2019 (n=125) e 2020 (n=168).

As variáveis recomendadas para a autoapresentação *online* incluem variáveis individuais, cultura/filiação de grupo, motivações, variáveis específicas da mídia social, conteúdo de autoapresentação gerado por si mesmo e por outros, bem como a eficácia de auto apresentação.^15^

Os perfis foram analisados quanto ao tipo (pessoal ou institucional) e gênero. Verificou-se que 0,8% dos perfis analisados eram institucionais. Entre os perfis pessoais, 76,5% eram de homens e 21,2% de mulheres. Registrou-se ainda que para 1,5% das contas não foi possível identificar o gênero dos usuários das contas.

Além da análise quanto ao tipo do perfil, o estudo buscou mapear e categorizar as URLs disponibilizadas em cada uma das contas como possibilidade de informações adicionais ou de vínculo profissional dos usuários. Apenas 241 perfis disponibilizaram URL na descrição do perfil e a distribuição das URLs por tipo pode ser verificada no Gráfico 2.

Os benefícios do uso do Twitter (X) por médicos incluem melhoria na comunicação médico-paciente e médico-médico, promoção da saúde, rastreamento de tópicos em saúde e doenças e construção de identidade online positiva.^16^ Esses podem ser observados com uma atuação consistente e no uso de funcionalidades, como compartilhamento de conteúdos com link (URL) e interações com outros usuários da rede por meio de menções e respostas (@) e reprodução de conteúdo de terceiros (RT).

Os dados indicam que essas práticas são pouco realizadas pelos cardiologistas brasileiros no Twitter (X), uma vez que apenas 1,9% possuem perfis com URL, e mensagens com interações com RT e @ também só foram registradas em 1,7% das contas. A baixa participação em mídias sociais pode estar associada ao fato de alguns médicos relutarem em se envolver em comunicação *online* com seus pacientes ou suas comunidades devido a preocupações com leis de responsabilidade e privacidade.^17^

Quanto ao conteúdo dos *tweets* foi possível analisar as hashtags mais utilizadas pelas contas no período analisado. A análise de presença e atuação nas mídias sociais com fins acadêmicos e profissionais costuma se valer de métricas e indicadores de desempenho. Entre esses, há os indicadores de conectividade social que agrupam métricas que expressam o grau em que um usuário está conectado com o resto da comunidade científica ou profissional que o cercam, e mesmo com a sociedade em geral. Portanto, a conectividade social corresponde às interações usuário-usuário, medidas pelo número de seguidos e seguidores.^18^

Os resultados da pesquisa indicam que as contas possuem ao todo 418 312 seguidores e seguem 293 006 perfis, o que corresponde a uma média de 386 seguidores e 270 seguindo. A conectividade social das contas analisadas pode ser observada na Tabela 1, onde podemos observar que poucas contas alcançam mais de 2000 seguidores. Embora a média de seguidores seja maior que a de contas seguindo, no geral, as contas não parecem atrair muitos seguidores. A maior concentração é de perfis com até 100 seguidores (71%) e a menor concentração é de perfis com mais de 1000 seguidores (4%). Quando analisamos essa distribuição de contas seguindo notamos que contas que seguem até 100 perfis (48,0%) ou mais de 100 até 1.000 (47,8%) estão bem próximas. As poucas contas com mais seguidores é que são responsáveis por elevar o valor da média para cima. A mediana calculada de 169,5 seguidores confirma essa assimetria com relação à média, efeito que não é visto para o número de contas que eles seguem, cuja mediana de 323 é bem próxima à média.

**Tabela 1:** – Conectividade social das contas do Twitter (X)

Seguidores	Contas	%
0 - 100	770	71,1
101 - 1000	269	24,8
1001 - 2000	22	2,0
n > 2000	22	2,0
**Total**	**1083**	**100**

A atuação no microblog – mantendo-se regularidade nas postagens com conteúdos relevantes e o uso dos recursos de interação – contribui para um bom desempenho na rede que, por sua vez, reflete na autoridade social do perfil. A Tabela 2 demonstra a distribuição das contas analisadas pela autoridade social. Ao se atribuir uma escala de 1 a 100 à autoridade social, percebemos que as contas analisadas não apresentam um bom desempenho nesse indicador tendo em vista que 81,8% não ultrapassaram 25 pontos e 15% apresentaram até 50 pontos, ou seja, pouco mais de 97% das contas não superaram a metade da escala do indicador. Para qualificar um pouco mais “autoridade social”, listamos os 20 principais perfis com melhor desempenho nesse indicador (Tabela 3).

**Tabela 2:** – Autoridade Social das contas do Twitter (X)

Autoridade Social	Contas	%
0 - 25	886	81,8
26 - 50	168	15,5
51 - 75	28	2,6
n > 75	1	0,1
**Total**	**1083**	**100**

**Tabela 3:** – Perfis com maior autoridade social no Twitter (X)

Nome da conta	Gênero	Língua	Local da conta	Autoridade Social
MBittencourtMD	M	Inglês	São Paulo	77,2
josenalencar	M	Português	São Paulo	70,7
fabiovboas	M	Português	Bahia	66,1
evandrofilhobr	M	Inglês	Alagoas	62,3
fabioepm	M	Português	São Paulo	61,4
pabeda1	M	Inglês	Rio de Janeiro	61,2
flaviobessajr	M	Português	Paraíba	61,1
lilianigromaia	F	Português	São Paulo	60,6
drluizovando	M	Português	Mato Grosso do Sul	58,1
Lucas_P_Freitas	M	Português	Minas Gerais	57,4
estadocida	F	Português	São Paulo	56,8
InacioCamba	M	Português	Rio de Janeiro	55,9
fikkumamoto	M	Inglês	Paraíba	55,7
carlosF201634	M	Inglês	Minas Gerais	54,8
AdrianaSerpa1	F	Português	Pernambuco	54,6
Leticiagrocha_	F	Português	Rio de Janeiro	53,1
IMaranhao666	M	Português	São Paulo	52,9
DrSergioBarros	M	Inglês	São Paulo	52,5
brunobalencar	M	Português	Brazil	52,3
rauldsf_santos	M	Inglês	São Paulo	51,4

**Figura 3:**
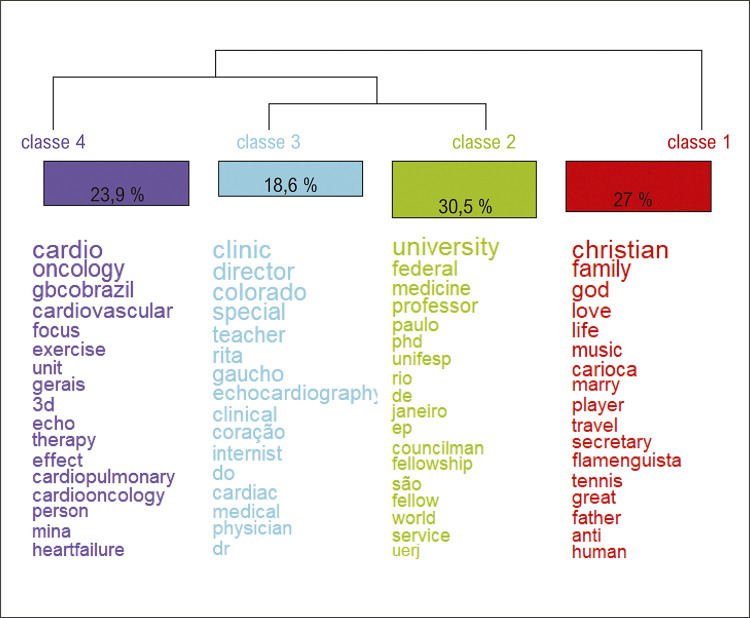
– Filograma gerado no Iramuteq mostrando as classes de palavras mais utilizadas por cardiologistas brasileiros em suas bios do Twitter (X).

Quanto ao conteúdo da autoapresentação, foi possível analisar os termos e expressões mais recorrentes na descrição da bio. Analisando o filograma na Figura 3, percebe-se que o conjunto de textos obtidos a partir dos Tweets analisados pelo programa foi dividido em dois eixos: um profissional e outro pessoal. O primeiro conjunto divide-se em três temáticas relacionadas ao âmbito profissional: a primeira (23,9%; azul escuro) em sua maioria está relacionada às especializações na área da saúde (Cardio-oncologia, Cardiologia), doenças cardiovasculares (insuficiência cardíaca, cardiopulmonar), exames e tratamentos relacionados principalmente a doenças do coração (eco, 3D, exercício, terapia) e referências a grupos de profissionais da área médica (gbcobrazil - Grupo Brasileiro de Cardio-Oncologia). Assim, o assunto central nessa classe foi a “prática profissional médica”, mais especificamente na área da cardiologia. A segunda (18,6%, verde-água) está mais voltada para os cargos que os usuários ocupam, como diretor, professor, interno, clínico, médico, entre outros. Trata-se de uma categoria que demarca a autoridade pelo exercício profissional. Já a terceira (30,5%, verde) refere-se à vinculação institucional, sobretudo universidades e demais instituições de pesquisa. Trazem palavras que remetem à “autoridade e vinculação profissional” dos usuários, como referências a instituições de ensino (Universidade, UNIFESP, UERJ), formação (Medicina) e títulos (Ph.D., fellow, conselheiro), bem como a profissões (professor universitário) e estados da região Sudeste do Brasil (Rio de Janeiro, São Paulo). Em relação ao eixo pessoal (27%, vermelho), as autodescrições estão relacionadas a interação entre “gostos e valores” dos usuários. Verifica-se menção a termos relacionados à religião (cristão, Deus, vida) e à família (casar, pai), bem como termos relacionados a atividades esportivas (jogador, tênis, flamenguista) e ao lazer (música, viagem). Percebe-se, pela análise de CHD (Figura Central), que mostra como as palavras se relacionam em grupo, que este eixo pessoal apresenta certo distanciamento em relação às demais.^14^

A AFC permite, por meio de gráficos, visualizar a proximidade, ou seja, as relações entre as palavras e das classes oriundas da CHD (Figura Central). A AFC veio confirmar as percepções já obtidas com a figura anterior. O eixo pessoal mostra-se mais isolado das outras, de modo que apenas algumas de suas palavras representativas se misturam às das outras classes. Já os grupos de palavras relacionados às filiações institucionais e aos cargos e atividades profissionais vigentes se encontram muito próximas, com grande parte de seus termos misturados entre si. Uma ligação entre esses grupos é plausível, já que, por exemplo, ambas incluem termos relacionados a profissões (professor, médico, internista) e títulos (Ph.D., Dr.).

## Discussão

Este estudo investigou o perfil dos cardiologistas brasileiros no Twitter (X), com foco em sua presença, influência e alcance *online*, bem como na apresentação de suas biografias. Os resultados revelaram algumas características importantes sobre a comunidade de cardiologistas no Twitter (X) no contexto brasileiro. Uma descoberta interessante foi que a maioria dos cardiologistas brasileiros, usuários do Twitter (X), opta por utilizar suas contas pessoais para se engajar na plataforma. Isso pode indicar uma preferência por uma comunicação mais pessoal e direta com seus colegas, pacientes e seguidores, em vez de uma representação institucional. Nakagawa et al.^19^ avaliaram o perfil dos 100 maiores influenciadores em cardiologia de 2016 até 2020 e observaram uma predominância de perfis de cardiologias dos Estados Unidos da América e Europa, e nenhum cardiologista da América Latina.

Dos 20 perfis de maior influência, a maioria era de homens (80%), com alta concentração na região sudeste (68%), refletindo o perfil da área da cardiologia no país. Interessante foi o crescimento significativo de contas de cardiologistas brasileiros no ano de 2009. Esse aumento acompanhou o crescimento no número global de usuários brasileiros do Twitter (X) que aumentou de 1 milhão em 2008 para 4 milhões em 2009. Esse crescimento foi impulsionado por uma série de fatores, incluindo o lançamento do Twitter (X) para dispositivos móveis em português e o aumento da popularidade da plataforma entre celebridades e influenciadores brasileiros.

Outra observação relevante é a disparidade de gênero entre os cardiologistas brasileiros no Twitter (X). A maioria (76,5%) dos perfis identificados como cardiologistas pertencia a homens, enquanto apenas 21,2% eram de mulheres. Esses dados reproduzem os achados do próprio Twitter (X) que mostraram que em 2022 cerca de 69% das contas eram de homens e 31% eram de mulheres. Essa disparidade é mais acentuada em algumas regiões do mundo, como no Oriente Médio e na África, onde as mulheres representam apenas 20% dos usuários do Twitter (X). É importante investigar mais a fundo as razões por trás dessa disparidade de gênero e explorar formas de promover uma maior participação e representação das mulheres cardiologistas na plataforma. Sarah e colaboradores avaliaram diversos sites e mídias sociais e encontraram disparidades importantes quanto ao gênero e etnia, reforçando a necessidade de maior compreensão sobre o tema.^20^

No que diz respeito ao alcance e influência online dos cardiologistas brasileiros no Twitter (X), observou-se que o número médio de seguidores por conta foi de 386, enquanto o número médio de perfis seguidos por conta foi de 270. Esses números indicaram um certo grau de interconexão e engajamento entre os cardiologistas brasileiros na plataforma. No entanto, também foi observado que a maioria das contas tinha um número relativamente baixo de seguidores e baixa autoridade social. Isso sugere que a influência *online* dos cardiologistas brasileiros no Twitter (X) ainda é limitada na maioria dos casos.

As porcentagens de contas com até 100 seguidores (71%) e a de contas com mais de 1000 seguidores (4%) indicam que a maioria dos cardiologistas brasileiros no Twitter (X) tem um alcance relativamente limitado. Isso pode ser atribuído a vários fatores, como a natureza específica do campo da cardiologia e a competição com outros especialistas e conteúdos na plataforma.

Essas descobertas podem refletir desigualdades existentes no campo da cardiologia, incluindo disparidades de gênero e desigualdades regionais no acesso a oportunidades e recursos. Estudos recentes sugerem que as mídias sociais como o Twitter (X) podem ser ferramentas efetivas para disseminar informações e inovações médicas e aumentar a produtividade acadêmica.^21^ Isto deve ser levado em conta pelos usuários cardiologistas como forma de ampliar o alcance de suas atividades.

O uso do Twitter (X) por cardiologistas brasileiros apresenta desafios significativos. Primeiramente, a baixa autoridade social de suas contas pode ser atribuída a vários fatores. A barreira da língua portuguesa pode limitar a visibilidade internacional, uma vez que grande parte do conteúdo científico é compartilhado em inglês. Além disso, o número reduzido de publicações produções científicas na área cardiovascular no Brasil, em comparação a outros países presentes na rede social, também afeta a credibilidade e o alcance das contas dos cardiologistas brasileiros. Para superar essas limitações, é crucial incentivar a participação ativa desses profissionais no Twitter, promovendo a disseminação de conhecimento e colaborações internacionais.^20^

Uma limitação importante do nosso estudo é que utilizamos um corte temporal restrito para análise das contas do Twitter (X). Esta rede social tem passado por modificações constantes ao longo do tempo que podem haver afetado a participação dos cardiologistas brasileiros, apesar de acreditarmos que os usuários médicos não tenham sofrido impacto significativo das mudanças. Uma vez que a coleta de dados se baseou na autoapresentação, a pesquisa pode apresentar limitações de cobertura devido a não identificação de cardiologistas que não se apresentam na plataforma como tal. A busca por temas, expressões ou *hashtags* que denotam debates na área de Cardiologia como o #CardioTwitter pode contornar esse tipo de limitação e complementar as contas identificadas. Por fim, não foi realizada uma busca por perfis institucionais e, consequentemente, a baixa taxa de 0,8% desses perfis identificados era esperada. Assim, essa limitação deve ser considerada na interpretação dos resultados do estudo.

## Conclusão

Os cardiologistas brasileiros com presença e atuação no Twitter (X) apresentaram uma baixa autoridade social, que pode em parte, ser explicada pelo uso da língua portuguesa nas suas publicações. Observamos uma disparidade de gênero entre os cardiologistas brasileiros na plataforma, com predominância de homens. Os perfis com maior influência *online* foram de homens, sendo identificada uma alta concentração de usuários na região sudeste. Novos estudos devem ser realizados sobre o tema para verificar o impacto dessas características na população ao longo do tempo.

**Figura 1:**
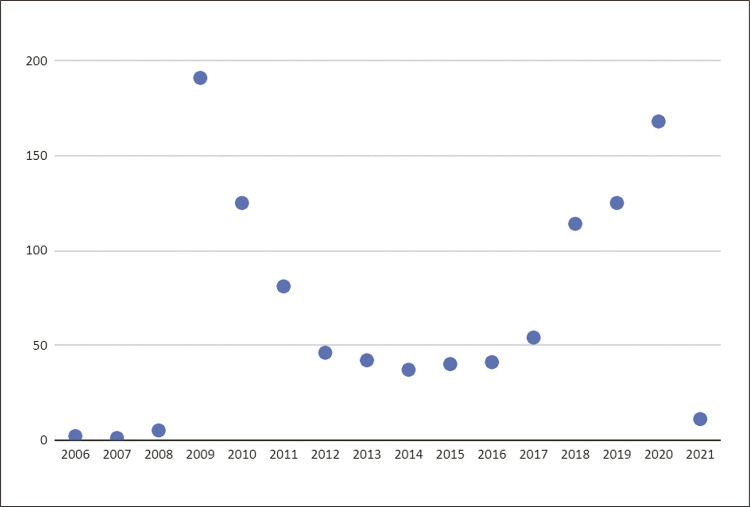
– Distribuição das contas do Twitter (X) pelo ano de criação.

**Figura 2:**
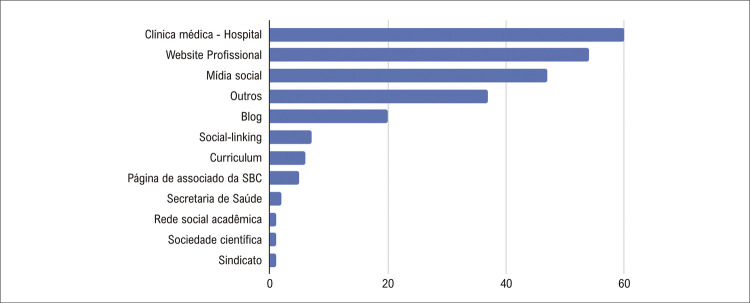
– Análise das URLs a partir das bios dos perfis das contas do Twitter (X) de cardiologistas brasileiros.
