# Supplementary material for: O Twitter (X) como Ferramenta de Comunicação e Educação para Cardiologistas Brasileiros: Perfil, Influência e Desafios
Source: Arq Bras Cardiol. 2024 Nov 27;121(12):e20230694. [Article in Portuguese] doi: 10.36660/abc.20230694 (PMC11634314; doi:10.36660/abc.20230694)
Supplement: Translation

# Translation: Twitter (X) as a Communication and Education Tool for Brazilian Cardiologists: Profile, Influence and Challenges

**DOI:** 10.36660/abc.20230694i

**Authors:** Aline Goneli de Lacerda, Luana de Oliveira Ribas, Estephany de Jesus, Ronaldo Ferreira de Araújo, Thaiane Moreira de Oliveira, Claudio Tinoco Mesquita

**Affiliations:** 1 Brazil Universidade Federal Fluminense, Niterói, RJ – Brazil; 2 Brazil Universidade Federal de Alagoas, Maceió, AL – Brazil

**Keywords:** Online Social Networking, Cardiologists, Health Communication

## Abstract

**Background:**

Twitter (recently renamed to X) is a popular social media that can be used for health communication. There are few studies analyzing the profile of Brazilian cardiologists active on the platform.

**Objectives:**

To identify the profile of Brazilian cardiologists on Twitter (X), their online influence and reach, as well as their bios’ description.

**Methods:**

A total of 1,083 accounts of Brazilian cardiologists were created between 2006 and 2021. Data were collected using the FollowerWonk tool and analyzed using the IRAMUTEQ software.

**Results:**

Most profiles were of men (76.5%) and 0.8% was institutional profiles. Most profiles had less than 100 followers (71%) and low social authority (81.8%). The 20 most influential profiles were mostly of men (80%) and highly concentrated in the southeast of Brazil (68%).

**Conclusions:**

Brazilian cardiologists prefer a personal and direct communication in social medias, rather than an institutional representation. There is a gender disparity among Brazilian cardiologists on Twitter (X), with a predominance of men. The most influential profiles were of men, and mostly located in the southeastern Brazil.

## Introduction

Since Internet has become a common way by which people disseminate and access information about health,^1^ social medias has been an important place where health professionals share study results and scientific data and strengthen their relationships with patients.^2^

Twitter (X) is currently the most popular social media used for communication in health.^3^ Sharing information on Twitter (X) may create a collaboration and communication environment among patients, physicians and researchers, and even improve the quality of healthcare. Due to the resources available in the platform that allow an interpersonal conversational communication,^4-6^ the tweets can capitalize on social media to amplify the coverage of health messages.^7^

Twitter (X) has gained importance as an academic forum, particularly for its microblogging nature, that allows direct, rapid, real-time interactions among specialists.^8^ Nearly 20% of the articles published in Pubmed are tweeted at least once, which increases the likelihood of citation.^5^ However, despite this scientific achievement, few physicians and scientists engage in Twitter (X) routinely,^9^ as indicated in a study reporting that only 238 (16%) of 1,500 cardiologists had a Twitter (X) account. Although there are several potential explanations for his relatively low adherence to Twitter (X) among scientific community, important issues related to unreasonable points of view, data manipulation, inefficient use of time and patient privacy are probably contributing factors.

As observed by Caleb Fergunson et al.,^10^ the percentage of professionals in the cardiovascular field (journals and associations) that use Twitter (X) to interact with other people and exchange ideas has increased. The assessment of the scope and the impact of research on health and clinical practice in social medias may provide information on the best strategies to foster the use of social networks. Although some authors have discussed the professional profile of researchers and healthcare providers in social medias in different countries,^3,10,11^ so far, there are no studies focused on the Brazilian context.

Therefore, the aim of the present study was to identify Brazilian cardiologists among Twitter (X) users, analyze their impacts and reach, as well as their bios’ description. We believe that the use of digital medias by cardiologists is a way to construct the social authority and capital required to understand how the field of Cardiology could be presented in the microblog.

## Methods

This is an exploratory study, with a quantitative and descriptive approach aimed at identifying the online presence, visibility and influence of Brazilian cardiologists on Twitter (X).

### Data collection

Twtitter (X) profiles (bios) of the users were examined using a web-based tool Followerwonk (https://moz.com/followerwonk) and the keywords ‘cardiologist’ OR ‘cardiologista’ in December 2022. Followerwonk can visualize Twitter (X) networks geographically, compare different user accounts, and analyze the content of Tweets from particular regions.^12^

All data extracted from the profiles, including the Social Authority Score (SAS), were exported to a database spreadsheet for descriptive statistical analysis. The SAS is a Twitter (X) influence scale (1–100) that considers key performance indicators such as number of followers, user mentions, number of retweets and engagement of the users’ publications on Twitter (X).^13^

Exclusion criteria of the profiles were: (a) personal or institutional profiles that did not belong to cardiologists; (b) Twitters accounts written in languages other than Portuguese or English, (c) inactive user (no posts in the last six months); (d) user’s location outside Brazil or the user is not affiliated to a Brazilian institution; (e) restricted accounts; and (f) profiles with no photos.

### Data analysis

The variables considered in the analysis were: (i) number of profiles identified as Brazilian cardiologists and date of account creation; (ii) URLs available in the profile description; (iii) number of followers of Brazilian cardiologists (mean, standard deviation); (iv) top 100 social authorities; (v) correlation of the 100 main geographic locations and social authority; (vi) gender and race inequality related to the use of cardiology on Twitter (X), and (vii) the most common topics tweeted.

Data from the bio description of each user were extracted and organized in a csy spreadsheet. For data processing, the software IRAMUTEQ (*Interface de R pour lês Analyses Multidimensionnelles de Textes et de Questionnaires*) was used; IRAMUTEQ is a free software based on the R language that enables processing and statistical analysis of the texts.^14^ Analysis of the textual content retrieved from the bios was made by the Descendant Hierarchical Classification (DHC) and the Correspondence Factor Analysis (CFA), which allow its identification via a single txt file, properly configured.

## Results

Descriptive data extracted from the Brazilian cardiologists’ profiles identified on Twitter (X) revealed that the 1083 accounts analyzed were created between 2006 and 2021. Graph 1 illustrates the distribution of accounts by year of creation.

There was a low adherence to Twitter during the first years of the microblog, with the highest number of accounts created in 2009 (n= 191) and 2010 (n=125), followed by a progressive decrease in the subsequent years. In 2017 onwards, the number of accounts started to increase again, particularly in 2019 (n=125) and 2020 (n=168).

The variables suggested to be used in online self-presentations include individual traits, group membership, motives, specific social media variables, self-presentation content and feedback from others, as well as the efficacy of self-presentation.^15^

The profiles were analyzed in terms of type (personal or institutional) and gender; 0.8% were institutional profiles, and among personal profiles, 76.5% were men, 21.2% were women, and in 1.5% of the accounts the gender could not be identified.

In addition, the study sought to map and categorize the URLs available in the accounts to check for additional information of occupation of the users. The URLs were available in only 241 of the profiles, and their distribution by type is illustrated in Graph 2.

The benefits of physician participation in Twitter (X) includes communication enhancement (physician-patient and physician-physician), health promotion, screening for health and disease-related topics, and construction of a positive online identity.^16^ These can be achieved by consistent engagement and use of functions by the users, like sharing of contents with links (URL), and interactions with other users by mentions, @replies and retweets.

However, the data indicated that these practices are not often performed by Brazilian cardiologists on Twitter (X), since only 1.9% have a Twitter profile with URL, and messages with interactions with retweets and @ were seen in only 1.7% of the accounts. The low participation in social medias among physicians may be associated with the fact that some professionals may be reluctant to engage in online communication with their patients or their communities due to responsibility- and privacy-related issues.^17^

With respect to the content of the tweets, we analyzed the most used hashtags in the accounts during the study period. Analysis of the presence and engagement in social medias with academic or professional purposes is usually made by metrics and performance indicators. These indicators include social connectivity which groups metrics that express the extent to which a user is connected with the rest of the scientific or professional community that surrounds him, and even with the society in general. Therefore, it corresponds to user-user interactions, measured by the number of followers and followings.^18^

Results of the present study indicates that the accounts analyzed had a total of 418,312 followers and followed 293,006 profiles, corresponding to a mean of 386 followers and 270 followings. Social connectivity of these accounts is described in Table 1, showing that few of them reached 2,000 followers. Although the mean number of followers was higher than the number of followings, in general, the accounts seem not to attract many followers. Most profiles (71%) had up to 100 followers and only 4% had more than 1,000 followers. Analysis of the distribution of the following accounts revealed that the percentage of accounts following up to 100 profiles (48.0%) was similar to that of accounts following more than 100 and less than 1000 profiles (47.8%). The few accounts with the highest number of followers were responsible for increasing the average. The median number of followers was 169.5, which confirms this asymmetry around the mean; this effect is not seen with the number of accounts they follow, whose median was 323, *i.e*., very close to the mean.

**Table 1:** – Social connectivity of Twitter (X) accounts

Followers	Accounts	%
0 - 100	770	71.1
101 - 1000	269	24.8
1001 - 2000	22	2.0
n > 2000	22	2.0
**Total**	**1083**	**100**

The engagement on the microblog – regular postings containing relevant content and the use of interaction resources – contributes to a good performance on the Internet, which reflects in the social authority of the profile. Table 2 describes the distribution of the accounts by social authority. By assigning a 1-100 scale to social authority, we observed that the accounts had a poor performance in this indicator, since 81.8% scored less than 25 points and 15% less than 50 points, *i.e*., a bit more than 97% of the accounts did not score higher than half of the scale. To further quality “social authority”, we listed the 20 profiles with the best performance in this indicator (Table 3).

**Table 2:** – Social authority of the Twitter (X) accounts)

Social authority	Accounts	%
0 - 25	886	81.8
26 - 50	168	15.5
51 - 75	28	2.6
n > 75	1	0.1
**Total**	**1083**	**100**

**Table 3:** – Users’ profiles with the highest social authority on Twitter (X)

Name	Gender	Language	Place	Social authorit
MBittencourtMD	M	English	São Paulo	77.2
josenalencar	M	Portuguese	São Paulo	70.7
fabiovboas	M	Portuguese	Bahia	66.1
evandrofilhobr	M	English	Alagoas	62.3
fabioepm	M	Portuguese	São Paulo	61.4
pabeda1	M	English	Rio de Janeiro	61.2
flaviobessajr	M	Portuguese	Paraíba	61.1
lilianigromaia	F	Portuguese	São Paulo	60.6
drluizovando	M	Portuguese	Mato Grosso do Sul	58.1
Lucas_P_Freitas	M	Portuguese	Minas Gerais	57.4
estadocida	F	Portuguese	São Paulo	56.8
InacioCamba	M	Portuguese	Rio de Janeiro	55.9
fikkumamoto	M	English	Paraíba	55.7
carlosF201634	M	English	Minas Gerais	54.8
AdrianaSerpa1	F	Portuguese	Pernambuco	54.6
Leticiagrocha_	F	Portuguese	Rio de Janeiro	53.1
IMaranhao666	M	Portuguese	São Paulo	52.9
DrSergioBarros	M	English	São Paulo	52.5
brunobalencar	M	Portuguese	Brazil	52.3
rauldsf_santos	M	English	São Paulo	51.4

Regarding self-presentation, we analyzed the most used terms and expressions by the users in their bios’ descriptions. As shown in the phylogram in Figure 3, all the texts extracted from the tweets and analyzed by the software were divided into two axes – one professional and one personal axis. The first subgroup contains three professional-related subjects: the first one (23.9%; dark blue) refers mostly to specializations in the medical field (cardio-oncology, cardiology) cardiovascular diseases (heart failure, cardiopulmonary failure), exams and therapies used in cardiovascular diseases (echo, 3D, exercise, therapy) and references to medical groups (gbcobrazil - Grupo Brasileiro de Cardio-Oncologia). Thus, the core subject in this class was “medical professional practice”, more specifically in the cardiology field. The second subject (18.6%, in acqua blue) refers mostly to users’ occupations and work positions (director, teacher, intern, clinician, doctor, among others. Authority is here determined by professional practice. The third subject (30.5%, in green) refers to institutional affiliation, mostly universities and other research institutions. It includes words referring to “authority and professional affiliation” as references to educational institutions (University, UNIFESP, UERJ), graduation (Medicine) and titles (PhD, fellow, tutor), job positions (lecturer) and states in the southeast region of Brazil (Rio de Janeiro, São Paulo). With respect to the personal axis (27%, in red), self-descriptions are related to the “preferences and values” of the users. Words related to religion (Christian, God, life), family (get married, father), and others related to sports (player, tennis, Flamengo supporter) and hobbies (music, travel) were used in this category. Analysis of the DHC (Central Illustration), which outlines the relationship of the word clusters, revealed that this personal axis is more distant from the other axes.^14^

**Figure 3:** – Phylogram generated by Iramuteq showing the most used words by Brazilian cardiologists in their Twitter (X) bios, by class.

The CFA allows, through graph analysis, to visualize the proximity (*i.e*., the relationship) between words and classes retrieved from the DHC (Central Illustration). The CFA corroborated the insights from Figure 3. The personal axis is more isolated from the others, and only a few of its representative words were mixed with words of other classes. On the other hand, the words related to institutional affiliations and current job positions were very close to and mixed with each other. It is plausible to suggest a link between these groups since both include work-related words (teacher, doctor, intern) and titles (PhD, Dr.).

## Discussion

The present study investigated the profile of Brazilian cardiologists, active users of Twitter (X) in terms of presence, influence, online reach, and bios’ description. The results revealed some relevant features of the cardiologist community on Twitter (X) in Brazil. One interesting finding was that most Brazilian cardiologists, users of Twitter (X), use their personal accounts to engage in the platform. This suggests a preference for a more personal and direct communication with their colleagues, patients and followers, over an institutional representation. Nakagawa et al.^19^ evaluated the profile of the top 100 influencers in the cardiology field between 2016 and 2020 and found a predominance of cardiologists from the USA and Europe, and there was no Latin-American cardiologist on the list.

Of the 20 most influential profiles, most were men (80%) and located in the southeast region, reflecting the profile of cardiology in the country. An interesting finding was the increase in the number of accounts of Brazilian cardiologists created in 2009. Such increase accompanied the growth in the number of Brazilian users of Twitter (X), which increased from one million in 2008 to four million in 2009. This phenomenon was triggered by several factors, including the launch of Twitter (X) for mobile devices in Portuguese and the greater popularity of the platform among Brazilian celebrities and influencers.

Another notable observation is the gender disparity among Brazilian cardiologists on Twitter (X). Most (76.5%) of the profiles identified as belonging to cardiologists belonged to men whereas 21.2% to women. These findings reproduce the distribution of Twitter (X) profiles in 2022, with nearly 69% of the accounts belonging to men and 31% to women. Such disparity is more pronounced in some regions of the world, like the Middle East and Africa, where women account for only 20% of Twitter (X) users. It is important to further investigate the reasons for such gender disparity and to explore ways to promote greater participation and representation of female cardiologists on Twitter (X). Sadah et al. evaluated several websites and social medias and found marked disparities of gender and ethnicity, reinforcing the need for a deeper understanding about the theme.^20^

With regards to online reach and influence of Brazilian cardiologists on Twitter (X), we found that the mean number of followers was 386 per account, while the mean number of profiles followed per account was 270. These numbers indicated some degree of interconnection and engagement among Brazilian cardiologists on the platform. However, we also observed that most of the accounts had a relatively low number of followers and low social authority. This suggests that the online influence of Brazilian cardiologists on Twitter (X) is still limited in most cases.

The percentages of accounts with less than 100 followers (71%) and of accounts with more than 1,000 followers (4%) indicated that most Brazilian cardiologists on Twitter (X) had a relatively low reach. This may be attributed to several factors, including the nature of the cardiology field and the competition against other specialists and contents on the platform.

These findings may reflect inequalities in the field of cardiology, including gender and geographic disparities regarding the access to opportunities and resources. Recent studies have suggested that social medias like Twitter (X) can be effective in disseminating information and medical innovations, and in leveraging academic productivity.^21^ This should be considered by cardiologists willing to broaden the reach of their activities.

Brazilian cardiologists face important challenges in using Twitter (X). First, the low social authority of their accounts may be attributed to several factors. First, Portuguese language may be a barrier to international visibility, since a massive part of scientific information is shard in English. Besides, the reduced number of scientific publications and productions in the field of cardiology in Brazil, as compared with other countries active on social medias, also affects both credibility and reach of Brazilian cardiologists’ accounts. To overcome these limitations, it is crucial to encourage the active participation of these professionals on Twitter, to foster knowledge dissemination and international collaboration.^20^

One important limitation of this study was the fact that we applied a restricted period for analysis of the Twitter (X) accounts. Twitter (X) has suffered several changes over time that may have affected the participation of Brazilian cardiologists, although we believe that this

was not the case. Since data collection was based on self-presentation, the study may have limitations in not identifying cardiologists who did not self-present as such on Twitter (X). A search for subjects, expressions or hashtags denoting debates in Cardiology, for example #CardioTwitter, may overcome this limitation and complete the number of cardiologists’ accounts. Finally, we did not search for institutional profiles in the study, and hence the low percentage (0.8%) of these profiles in the results was expected. Therefore, this limitation should be considered in the interpretation of the results.

## Conclusion

Brazilian cardiologists identified as active users on Twitter (X) had a low social authority which may be partially explained by the use of Portuguese language in their publications. There was a gender disparity among the accounts, with predominance of men. The most influential profiles were also of men, and there was a high concentration of users in the southeast of Brazil. Further studies are needed to verify the impact of these features on the population over time.

**Figure 1:** – Distribution of Twitter (X) accounts by year of creation.

**Figure 2:** – Analysis of the URLs available on Twitter (X) bios of Brazilian cardiologists.
